# Dynamical mesoscale model of absence seizures in genetic models

**DOI:** 10.1371/journal.pone.0239125

**Published:** 2020-09-29

**Authors:** T. M. Medvedeva, M. V. Sysoeva, A. Lüttjohann, G. van Luijtelaar, I. V. Sysoev

**Affiliations:** 1 Saratov Branch of Kotel’nokov Institute of Radioengineering and Electronics of Russian Academy of Sciences, Saratov, Russia; 2 Institute of Higher Nervous Activity and Neurophysiology of RAS, Moscow, Russia; 3 Yuri Gagarin State Technical University of Saratov, Saratov, Russia; 4 Institute of Physiology I, Westfalische Wilhelms University, Münster, Germany; 5 Donders Centre for Cognition, Radboud University, Nijmegen, the Netherlands; 6 Saratov State University, Saratov, Russia; Sorbonne Universite UFR de Biologie, FRANCE

## Abstract

A mesoscale network model is proposed for the development of spike and wave discharges (SWDs) in the cortico-thalamo-cortical (C-T-C) circuit. It is based on experimental findings in two genetic models of childhood absence epilepsy–rats of WAG/Rij and GAERS strains. The model is organized hierarchically into two levels (brain structures and individual neurons) and composed of compartments for representation of somatosensory cortex, reticular and ventroposteriomedial thalamic nuclei. The cortex and the two thalamic compartments contain excitatory and inhibitory connections between four populations of neurons. Two connected subnetworks both including relevant parts of a C-T-C network responsible for SWD generation are modelled: a smaller subnetwork for the focal area in which the SWD generation can take place, and a larger subnetwork for surrounding areas which can be only passively involved into SWDs, but which is mostly responsible for normal brain activity. This assumption allows modeling of both normal and SWD activity as a dynamical system (no noise is necessary), providing reproducibility of results and allowing future analysis by means of theory of dynamical system theories. The model is able to reproduce most time-frequency changes in EEG activity accompanying the transition from normal to epileptiform activity and back. Three different mechanisms of SWD initiation reported previously in experimental studies were successfully reproduced in the model. The model incorporates also a separate mechanism for the maintenance of SWDs based on coupling analysis from experimental data. Finally, the model reproduces the possibility to stop ongoing SWDs with high frequency electrical stimulation, as described in the literature.

## Introduction

Absence epilepsy is characterized by recurring paroxysmal seizures with a diminishment of responsiveness and awareness with sudden onset and termination. Usually it starts to occur in children between the ages of 5 and 12 and it often spontaneously disappears at puberty or adolescence. Absences begin without an aura, and their duration rarely exceeds 10–30 seconds. The seizures are accompanied by a typical bilateral symmetrical and generalized 2.5 to 4 Hz spike-wave discharge (SWD) in the electroencephalogram (EEG).

The fact that spike-wave activity concomitant to the absences occurs rather suddenly and synchronously in all electrodes in the surface EEG, made the first researchers mistakenly believe that the absence pathogenesis has an origin in “deep” subcortical structures such as higher brain stem or thalamus, and that the pathological activity diverges from there to both hemispheres [1]. For more detailed studies towards the mechanisms of SWD generation, various animal models have been used; most well known are two genetic absence models, rats of the WAG/Rij strain [2] and Genetic Absence Epileptic Rats form Strasbourg (GAERS) [3]. These models, first discovered in the eighties of the previous century, are well documented and validated as models for childhood absence epilepsy. They show the for absence epilepsy characteristic clinical concomitants accompanying the SWDs, such as mild facial myoclonus, accelerated breathing, and twitching of the vibrissae [4], as well as many other characteristics contributing to face, predictive and construct validity [2].

Computational models on SWD generation made a great contribution to our understanding of the pathophysiology of absence epilepsy [5–7]. Currently there are a number of models that are quite distinct from each other including phenomenological [8] and biophysical models [9–11]; thalamic [12], cortical [13, 14] and thalamo-cortical [15], micro- [12, 16], meso- [17–21] and macroscale [7, 16, 22–28] models were described. Most models aim to reproduce the characteristics of SWDs, but some of them focus on effect of abnormal activity in thalamo-cortical networks, most often the thalamus was considered as the initiation site for SWDs [29–30]. These different models show that there is a basic understanding about the contribution of various types of intrinsic currents and synaptic receptors, and of the physiological conditions under which synchronized activity in the form of sleep spindles and SWDs may occur, including the necessary feedback of thalamus to cortex and vice versa and the role of the four key elements comprising the cortical pyramidal cells and interneurons, the thalamo-cortical relay cells, and neurons of the reticular thalamic nucleus.

It is hypothesized that the ability to generate SWDs, which develops with age, is a relatively small pathology in the matrix of connections in the brain, caused by genetic factors. Widespread neural mass models are able to reproduce significant number of characteristics of epilepsy, but they are limited in three points. First, they do not allow reproducing the effect that relatively small changes in the connectivity matrix are responsible for SWDs, since they have only one equation [18] or a small set of equations [7] for each cell type. Second, the interconnections inside the brain structures, e. g. inside the reticular thalamic nucleus and inside the cortex cannot be included, but they are known to be significant for the ability of SWD generation. Third, there is no possibility to model the deviation of the disease over the population, since only one set of parameters corresponds to the pathological conditions (in network models this is possible by varying the connectivity matrix).

Since there could be a lot of different models for the same observed phenomenon such as SWD, one needs some tool for model verification. Here, we propose the following criteria: 1) spectra, 2) mean duration and distribution of SWD duration, 3) response to external stimulation, 4) Granger causality analysis of connectivity. We propose to consider the model suitable if it reproduces all these characteristics of experimental data qualitatively and/or quantitatively.

The peculiarity of the current work is that it uses a combination of mathematical modeling methods from the first principle (direct modeling) and model methods by solving an inverse problem (inverse modeling, [31]), comparing the results obtained from experimental data from the GAERS and WAG/Rij models to our mesoscale model. The main idea of creating this model was the assumption that the network structure plays a major role in generating highly synchronized activity in cortical and thalamic neurons, and that this activity is a function of the entire network organization, not only of the focal area. This idea was already illustrated in general for integrate-and-fire model neurons in [32]. Modeling different brain structures of C-T-C networks with large ensembles of oscillators (of a spatially distributed system) was performed by Proske et al. in [33] for thalamocortical dysrhythmia and by Nuidel et al. in [29] for image processing. However, these works did not focus on representing properties of absence seizures and SWDs. The results by Rothkegel et al. [34] suggest that initiation and termination processes for epileptic seizures can be generated in specially organized neural networks.

Recently a first version of mesoscale phenomenological, C-T-C model for SWDs was proposed [35]. The aim of the current paper is to fix a number of the disadvantages of the previous model and to provide new features mirroring the physiological knowledge about SWDs and C-T-C networks in general. So, the objectives of the current paper are as follows:

1. to consider the relevant to the SWD generation part of thalamus to be split into ventroposteromedial thalamic nucleus (VPM, TC-nodes) and the inhibitory reticular thalamic nucleus (RTN, RE-nodes), while previously the thalamus was considered as a single compartment;
2. to consider two types of cortical cells: pyramidal (PY) cellsand interneurons (IN) in ratio 4:1, while previously interneurons were not taken into account;
3. to make difference between inhibitory and excitatory projections; in particular in the current model the nodes corresponding to cortical interneurons and RE-cellsprovide inhibitory projections to other nodes, including projections to other nodeswithin the same structure, while the projections of the nodes corresponding to PY and TC-cells are excitatory; previously there was no separation between excitatory and inhibitory couplings;
4. to provide both normal and pathological dynamics by means of coupling architecture; noise must not be necessary for obtaining normal dynamics, so all the computational experiments become completely reproducible and no source of external complexity would be assumed; to achieve this both thalamic and cortical parts of the network are split into the focal and surrounding (larger) area, which is also partly involved in the generation of SWDs.
5. to provide three different ways to initiate SWD: due to increase in intracortical excitability, by external input from nervus trigeminus [36], and low frequency modulation [37, 38], while previously only an external input was considered;
6. to obtain mean seizure length close to the length reported in experiments, including a seizure maintenance process [39];
7. to reproduce thereported in the literature [40] mechanism of seizure termination by means of high (130 Hz) frequency stimulation;
8. to reproduce the results of coupling analysis of experimental data, achieved by means of Granger causality [41, 42].

## Materials and methods

### Model structure

**Fig 1:**
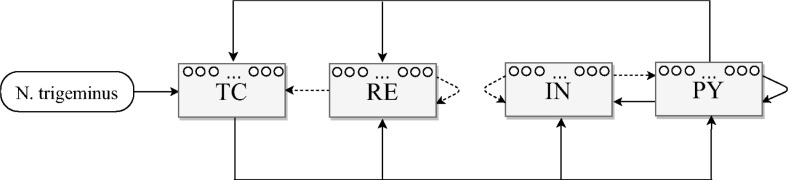
Coupling architecture of the proposed model. Inhibitory links are shown by dashed lines, exciting ones are solid. “PY” are pyramidal nodesin the cortex, “IN” are cortical interneurons, “TC” are thalamocortical nodes, “RE” are inhibitory nodesin the reticular thalamic nucleus, N. trigeminus serves as an external input.

Coupling architecture of the model was synthesized from the works [7, 18, 43] and others. It is illustrated in Fig 1. The model consists of four compartments: “PY” and “IN” for pyramidal cells and interneurons in cortex and “TC” and “RE” for thalamocortical cells and GABA-ergic reticular thalamic neurons in thalamus respectively. Further, to be able to compare model results to the experimentally observed phenomena, these compartments are also addressed as cortex (PY and IN together), VPM (ventroposterial medial nucleus of the thalamus, TC) and RTN (reticular thalamic nucleus, RE). Nervus trigeminus (N. trigeminus), innervating the whisker and frontal facial area, is considered as external input in the model and is modeled as an additional compartment.

Each compartment consists of a relatively high number of model nodesand is divided into two subpopulations. One, smaller subpopulation is modeling the focal areas in both thalamus and cortex. The number of model nodes in it is $N_{\mathrm{PY}}^{f}=40,\;N_{\mathrm{IN}}^{f}=10,\;N_{\mathrm{TC}}^{f}=40,\;N_{\mathrm{RE}}^{f}=40$. The other, larger subpopulation is modeling the surrounding areas. The number of model nodes in it is $N_{\mathrm{PY}}^{s}=160,\;N_{\mathrm{IN}}^{s}=40,\;N_{\mathrm{TC}}^{s}=80,\;N_{\mathrm{RE}}^{s}=80$. These surrounding areas correspond to other thalamic nuclei for TC and RE nodes (some of them can also be involved in SWDs, see e. g. [39, 44, 45]), and for cortical areas connected to somatosensory cortex (parietal cortex in the rat) for PY and IN nodes, including frontal and occipital cortex, which are normally involved in SWDs [3, 46]. The inclusion of these surrounding areas into the model was necessary to provide irregular oscillatory dynamics interictally. Therefore the total number of cells in each compartment was $N_{\mathrm{PY}}=N_{\mathrm{PY}}^{f}+N_{\mathrm{PY}}^{s}=200,\;N_{\mathrm{IN}}=N_{\mathrm{IN}}^{f}+N_{\mathrm{IN}}^{s}=50;\;N_{\mathrm{TC}}=N_{\mathrm{TC}}^{f}+N_{\mathrm{TC}}^{s}=120;\;N_{\mathrm{RE}}=N_{\mathrm{RE}}^{f}+N_{\mathrm{RE}}^{s}=120$. Additionally, giving *N*_NT_ = 10 nodes were used for n. trigeminus (“NT”), given 500 model nodes in total. All these numbers were set more or less empirically, based on our previous model [32], and to facilitate the occurrence of all necessary effect. The main conditions were the following: the number of interneurons being 4 times smaller than number of pyramid nodes, the total number of thalamic nodes should not be larger than the number of cortical ones.

The pathological part of the matrix does not work in isolation. And it is insufficient to reproduce interictal dynamics because it is small. Therefore, we also included the neurons of the surrounding areas of the same structures partially connected to the pathological matrix into the model. Otherwise, interictal activity would have to be modeled by noise as in [35]. But for the purpose of diagnosing external influences (stimulations), this approach is unacceptable, since the result will simultaneously depend on both the presence of noise and stimulus, so it will not be possible to identify the cause of the change in dynamics.

It is obvious that the number of model nodes is much smaller than the number of cells in the real brain. However, we were inspired by the idea from vacuum electronics, where billions of real electrons in reverse wave lamp are substituted in the model neither by the similar number of model electrons, nor by a single particle, but by some sufficiently large number of model “enlarged particles” [47], each of those is considered instead of large number of real particles with similar parameters. In electronics this approach allows to take into account electrical interactions between electrons in the tube, otherwise only interactions between the walls of the lamps and electrons and between electrons and the field are considered. In our case, model neurons (nodes) are analogous to these “enlarged particles”. On the one hand, such an approach makes it possible to simulate model time series using ordinary modern computers, since the number of network nodes is not too large. On the other hand, it allows to represent the essential interactions between nodes in the same structure (internal connections) in the model in contrast to neuron mass models like [7], where internal connections are represented by some effective parameters. Due to the described properties of the model, we call it *dynamical mesoscale model* (DMM) in comparison to the previous *stochastic mesoscale model* (SMM) described in [35], where interictal dynamics were not possible without noise.

### Model equations

Since network effects are the main theme of this study, the simplest possible neuron model was used for an individual node, that is FitzHugh-Nagumo [48, 49] Eq (1) with sigmoid coupling function *h*. While different models are known to be possible for the representation of individual neurons (see [50] for the most recent review), this one is one of the simplest oscillatory ones. Due to the presence of time delay *τ* in the model (1), they were solved using Euler algorithm, sampled with a step 0.5, and then time was renormalized in the ratio 1/1700, giving a sampling frequency *f*_samp_ = 3400 Hz to provide the time scale fitting the properties of experimental data; a similar effect was achieved by changing model parameters and coupling coefficients [35], but in that paper the parameter values had to be set with values far from essential ones reported in the literature.

$$\begin{array}{c} \frac{dx_{i}}{dt}=x_{i}\left(a-x_{i}\right)\left(x_{i}-1\right)-y_{i}+\sum_{j\neq i}C_{i,j}h\left(x_{i}\left(t-\tau \right)\right),\\ \frac{dy_{i}}{dt}=bx_{i}-\gamma y_{i},\\ h\left(x\right)=1+\frac{tanh\left(x\right)}{2}, \end{array}$$ (1)

Where *C* is a coupling matrix, and parameters of individual nodes set to be equal for all nodes *a* = 0.8, *b* = 0.008, *γ* = 0.0033. Time delay *τ*, corresponding to the time of synaptic conductance between nodes, was different for different matrices and was set to be 9–13 data points (approximately 2.6–3.8 ms in renormalized time).

The coupling matrix *C* entirely determines the dynamics of the network. The links between individual nodes were generated randomly, but following the scheme plotted in Fig 1 for couplings between brain structures and cell types. The coupling matrix generation was organized as follows. First, the whole matrix *C* was set to zero. Then couplings as presented in Fig 1 were set nonzero with different probabilities for different connections (see Table 1), with nodes in the focal area being coupled more often than in the surrounding ones. Values in Table 1 were fitted empirically in order to facilitate the occurrence of SWDs in the focal area. Only two nonzero values of *C*_*i*,*j*_ were used: 0.1 for excitatory couplings and −0.1 for inhibitory ones. At the final step, the collateral couplings were changed to the same (nonzero) value. The matrices for the focal area and the matrices for the surrounding areas were generated separately to simplify the process of further selection. In each case 7000 matrices were generated. Then, only matrices for the focal area being able to generate SWDs in response to short lasting increases in intracortical excitability (see the section “Onset due to gradual increase of intracortical excitability” for details) were selected. Also, matrices for surrounding areas generating chaotic dynamics without a well-established main frequency were selected. Then pairs of matrices for the focal area and for the surrounding areas were composed, under the condition that for both matrices in the pair the same value of *τ* was used. Matrices were coupled according to scheme (see Fig 1) and using probabilities for surrounding areas (see Table 1). As a result, four matrices with the desired properties were obtained.

**Table 1:** Probabilities of nonzero coupling between nodes in different compartments.

	Surrounding areas					Focal area				
*Node type*	PY	IN	TC	RE	NT	PY	IN	TC	RE	NT
PY	0.009	0.0315	0.0225	-	-	0.036	0.126	0.045	-	-
IN	0.009	0.0315	0.0225	-	-	0.036	0.126	0.045	-	-
TC	0.0135	-	-	0.01125	-	0.054	-	-	0.0225	0.18
RE	0.0135	-	0.0225	0.01125	-	0.054	-	0.045	0.0225	-

In Table 1 the probabilities of connections between nodes used for random model matrix generation are presented. Left half corresponds to the probabilities in the surrounding area submatrix, right half corresponds to probabilities in the focal areas.

Model time series for local field potentials (LFPs) were constructed as a sum of time series for all corresponding nodes. In particular, time series for the cortex were calculated as a sum of activities of all “PY” and “IN” nodes, model time series for the VPM were constructed as a sum of activities of all “TC” nodes, and model time series for the RTN were constructed as a sum of activities of all “RE” nodes.

### Experimental data

To compare the result of modeling with experimental data, two published datasets were used.

LPF recordings of the GAERS were used as experimental data, they were collected at the Institute of Physiology I, Westfälische Wilhelms Universität, Münster, Germany. All experimental procedures were carried out in accordance with the guidelines and regulations of the council of the European Union (Directive 2010/63/EU) and approved by local authorities (review board institution: Landesamtfür Natur, Umwelt und Verbraucherschutz Nordrhein-Westfalen; approval ID number: 84–02.04.2014.A398). Recordings of LPF from the somatosensory cortex (SI), the ventro-posterior medial nucleus of the thalamus (VPM) and the reticular nucleus of the thalamus (RTN) were used, recordings were performed in neurolept anesthetized rats. Data of this experiment were previously reported in [51].

LPF recordings of cortex and thalamus of symptomatic WAG/Rij rats were also used as experimental data. The data were collected at the Donders Center for Cognition, Radboud University, Nijmegen, the Netherlands. The experiment was approved by the Ethical Committee on Animal Experimentation of Radboud University Nijmegen (RU-DEC). LFP from free moving animals were from the somatosensory cortex (SI), the VPM and RTN. Data of this experiment were previously reported in [45].

## Results

### SWD initiation

Three ways of SWD onset were investigated in the model.

#### Onset due to gradual increase of intracortical excitability

**Fig 2:**
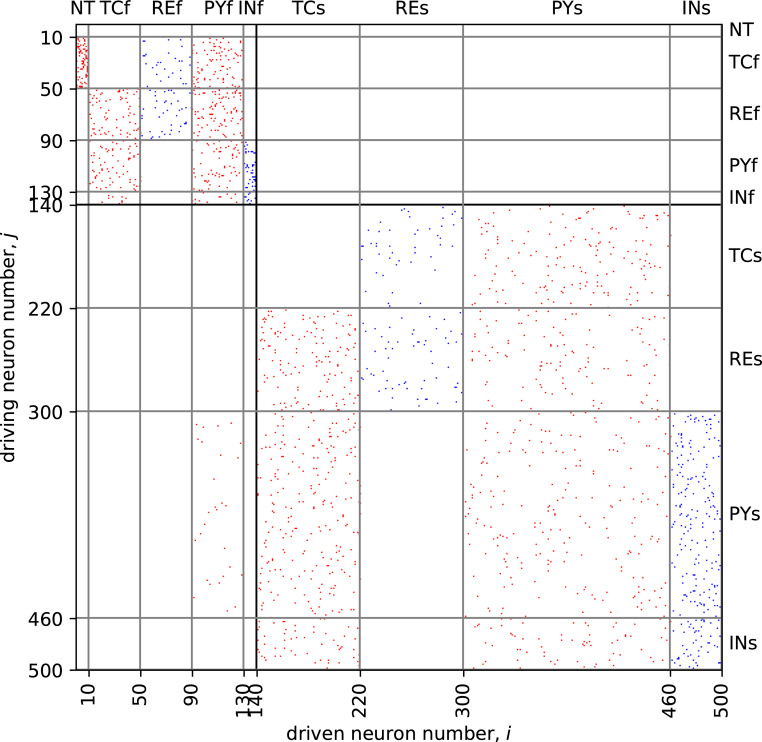
Connectivity matrix of the proposed model. Red points indicate the excitatory links and blue points represent inhibitory links. NT–Nervus trigeminus, TCf–thalamocortical nodes (focal area), REf–reticular thalamic neurons (focal area), PYf–pyramidal nodes (focal area), INf–interneurons (focal area); TCs–thalamocortical nodes (surrounding area), REs–reticular thalamic neurons (surrounding area), PYs–pyramidal nodes (surrounding area), INs–interneurons (surrounding area).

The onset of the SWDs was modeled by the simulation of processes of initiation of SWDs, as described earlier [39]. A short-term (0.3 s length) gradual preictal increase of the coupling coefficients (from the basic value 0.1 to the value 0.115) between PY neurons simulated the initiation process (see Fig 2, left column). One can see that SWD starts immediately in all channels after this temporary process has stopped, while during the initiation process LFP’s in all channels remain similar regarding its dynamics as interictally. This matches experimental findings from [39, 45], where the increase in intracortical coupling preceded the onset of SWD, while compared to preictal level a coupling decrease took place just after SWD onset.

#### Onset due to external driving from N. trigeminus

**Fig 3:**
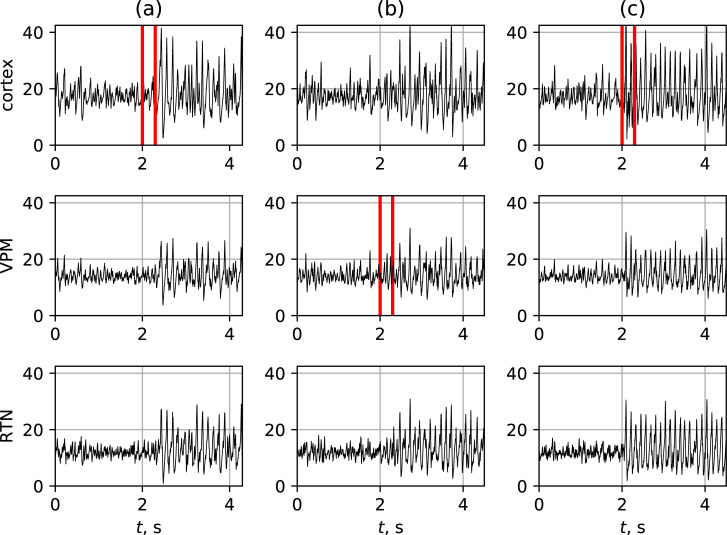
Three mechanisms of SWD onset. Model LFPs at SWD onset due to 3 different reasons: (a)–increase in intracortical excitability, (b)–external driving from N. trigeminus to VPM, (c)–low frequency harmonic stimulation of cortex. In all cases the initiation process starts at time moment 2 s and took place between the red lines.

Stimulation of peripheral nerves can result in SWD appearance as experimentally shown [36]. Here, following our first model [35] n. trigeminus, which has projections to the VPM and is therefore directly coupled to the thalamo-cortical system, is assumed as being a structure that is able to drive the VPM and somatosensory cortex. The short time (0.3 s) increase in coupling from n. trigeminus to VPM neurons from initial value 0.1 to the value 0.2 is shown in Fig 3B (the corresponding time interval is indicated between the red lines in VPM subplot). The spectral and shape characteristics of SWDs generated by means of this mechanism are the same as for SWDs generated by the increase in intracortical excitability. Moreover, all matrices selected to be able to generate SWDs using intracortical mechanism were able also to generate SWDs as a response to an external stimulus.

#### Onset due to low frequency stimulation

Luttjohann et al. in [52, 53] showed that low frequency cortical stimulation of the focal area in WAG/Rij rats can provoke SWDs. In the proposed model the discharge onset also can be elicited by using short-term stimulation applied to cortical PY neurons. Stimulation consisted of an 8 Hz sinusoid application to the PY and IN populations for 0.3 seconds. In order to simulate the stimulation, the second model equation in (1) was replaced by Eq (2) for cortical nodes (PY and IN) only.

$$\frac{dx_{i}}{dt}=x\left(a-x\right)\left(x-1\right)-y_{i}+\underset{j}{\sum }C_{i,j}h\left(x_{i}\left(t-\tau \right)\right)+sin\left(\omega t\right)$$ (2)

In contrast to SWDs initiated with increase of intracortical excitability, SWDs start in the time window of stimulation, not after. SWDs initiated with this mechanism are shown on Fig 3C. Their shape and amplitude do not differ from those of SWDs initiated by means of previously presented mechanisms in the same coupling matrix.

#### SWD maintenance

**Fig 4:**
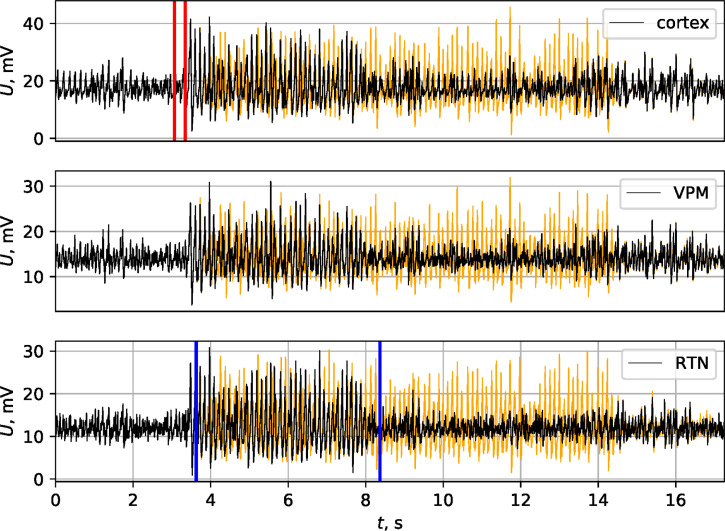
Effect of SWD maintenance process. Model SWD without separate maintenance process (black) and with maintenance (additionally increased coupling from “PY” nodes to “TC” nodes between blue lines). SWD was initiated by short in time increase of intracortical excitability (between red lines). Orange line shows the discharge with the maintenance process.

In [39] an increase in interactions between different thalamic nuclei (primary caudal RTN) and cortical layers based on results of coupling analysis was taken out. This coupling process was considered as a separate SWD maintenance mechanism which starts about 0.5–1.5 s after the onset of SWDs. This mechanism was included into the current model as relatively long coupling increase from RTN nodes to both pyramidal nodes and IN-nodes (see Fig 4). The coupling coefficients were increased from the normal value 0.1 to 0.115 for 5 s. This mechanism occurred to be very helpful for the model since its inclusion allowed both to support the signal amplitude during SWDs as well as their duration–see Fig 4. Most SWDs were elongated 1.5–3 times due to this mechanism, as shown by the orange curve in Fig 4 in comparison to the black one, while some of SWDs did not change.

### SWD termination

The mechanisms of SWD termination remain relatively unexplored. In [45, 41] no separate SWD termination mechanism was found and SWD termination was interpreted as a result of discontinuation of maintenance mechanisms. In [26] an increase in coupling from rostral RTN to cortex at 1 s before the SWD termination was detected and it was proposed to reflect or represent part of a possible SWD abortion mechanism, but this hypothesis needed more support. Therefore, no special mechanism of spontaneous SWD termination was provided in the current model.

#### Spontaneous termination

In the proposed model all SWDs terminated spontaneously. And this is also the case in the genetic models and in children with childhood absence epilepsy. The spontaneous termination was used as a criterion for the selection of the coupling matrices. In terms of nonlinear dynamics this means that in the current model each SWD is a long transient process rather than a stable regime. Such an approach is in agreement with modern ideas of nonlinear dynamics, with chimera states, heteroclinic trajectories and other transients rather than classical chaotic or regular attractors being considered as typical regimes of activity in models of neural networks [54–56].

**Fig 5:**
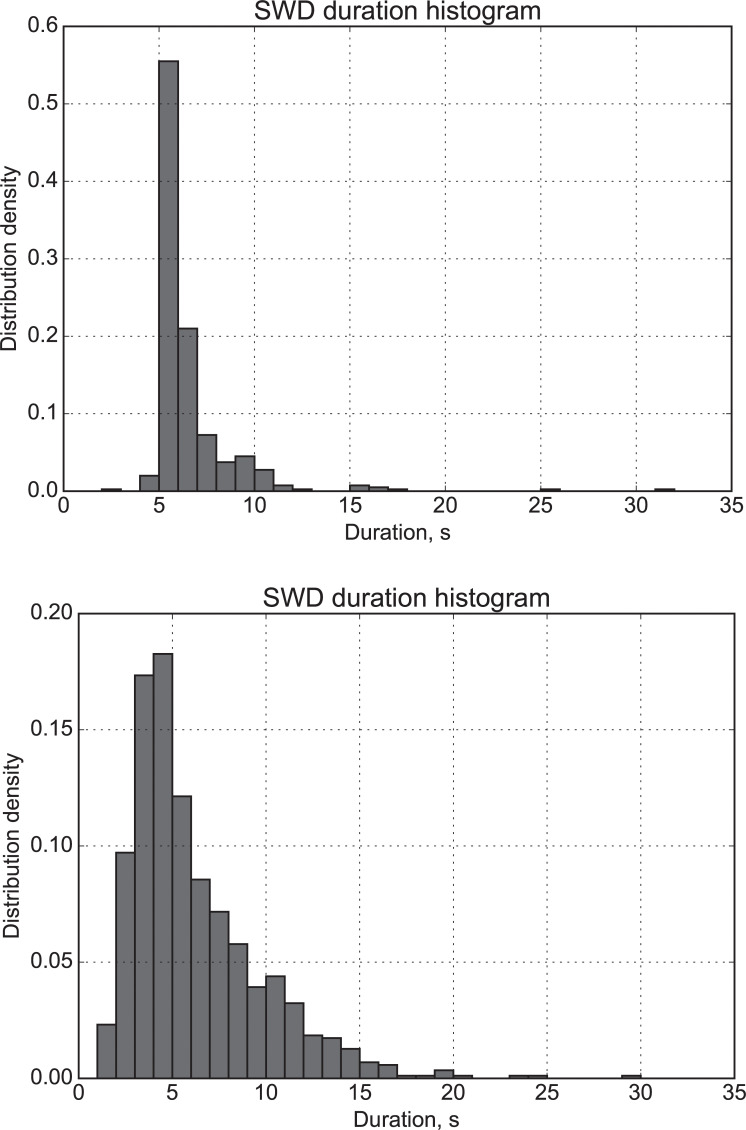
SWD probability density. Estimation of probability density of SWD duration of (a) calculated using 100 SWDs from 4 matrices, 400 in total, (b) calculated using LFP from WAG/Rij rats.

The distribution function of spontaneously terminated SWDs of the model (a) and real data (b) is shown in Fig 5. This distribution was constructed from 400 SWDs obtained from 4 matrices (100 per matrix). The maximum of distribution lies between 5 and 6 s which prettily matches the experimental results obtained in the WAG/Rij model, in GAERS the SWDs may last longer [57]. Also, more than 90% of SWDs are shorter than 10 s, with very long seizures of 15–32 s still being possible. It should be mentioned that without a separate SWD maintenance process described in section “SWD maintenance”, the distribution would be significantly shifted to shorter values.

#### Termination with high frequency stimulation

High frequency stimulation was shown to be the effective tool favorable for the abortion of SWDs. Stimulation for 1 sec with 130 Hz at low intensities, either in cortex, thalamus or brainstem quickly aborted SWDs, as it was shown in vivo [38, 40] in WAG/Rij rats, and at other locations, e.g. basal ganglia in GAERS [58–60].

The current model is able to reproduce this termination mechanism. An example of 130 Hz pulse stimulation is plotted in Fig 5. The stimulus was applied to the cortex (to all PY and IN nodes of the focal area) after 4s after SWD onset. The characteristics of pulses were as follows: pulse amplitude 1mV, pulse duration 0.6ms, interpulse interval 8ms, total length of stimulation 1s. The SWD, as it would develop without stimulation, is plotted in orange. It can be seen that SWD stopped during the application of the stimulus. We considered four different matrices and ten seizures for each matrix; it was found that 60% of SWDs were successfully terminated.

### Comparison of model series and spectra to experimental ones

The main frequency of SWDs decreases during the discharge from 5 to 3 Hz for humans. In rats of the GAERS strain, the main frequency also decreases during the discharge from 8 to 7 Hz. In rats of the WAG / Rij strain a sharper decrease in the main frequency is observed—from 11 to 8 Hz. The main SWD duration is 5–6 s for humans, about 15 s for GAERS rats and 5–8 s for WAG/Rij rats [61].

#### Comparison of model LFPs to LFPs of WAG/Rij rats

**Fig 6:**
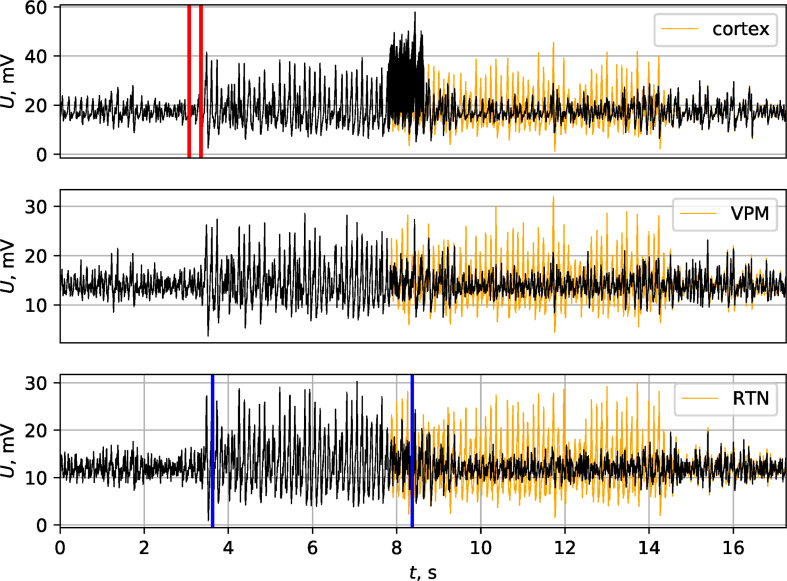
SWD termination by external high frequency stimulation. SWD was initiated by short in time increase in excitatory coupling between cortical nodes (time frame between two vertical red lines), and SWD maintenance process took place between two blue lines. Cortical 130 Hz pulse stimulation was started at time moment 7.5 s (about 4 s after seizure onset) and led to premature SWD termination. The stimulus can be seen in cortical model LFP at time moment 8 ± 0.5 s. The series, as it would develop without stimulation, is plotted with orange.

Typical time series and spectrograms of local field potentials at SWD measured from WAG/Rij rats are depicted in Fig 6 SWD start and termination are shown with black vertical lines. The increase in amplitude of the EEG signal during SWDs, in comparison to preictal and postictal states, is visible in plots of the LFPs. Also, the signals become more regular, the main frequency about 8.5 Hz and its higher harmonics (up to fourth one for cortex and VPM and up to third one for RTN) can be found in spectrograms during SWD. Amplitude of the cortical signal both during SWDs and interictally is higher than the amplitudes of signals from thalamic nuclei, and this is in agreement with what is commonly seen by us [46, 52] and might be due to the larger strength of the dipoles of cortical pyramidal versus thalamic neurons. Pyramidal cells are orderly oriented and have long, thick apical dendrites that can generate strong dipoles along the somatodendritic axis, and all this contributes substantially to the strength of the extracellular field. By contrast, thalamocortical cells, that have dendrites of relatively equal size in all directions, show only small dipoles and they contribute less to the extracellular fields responsible for the LPFs [62].

**Fig 7:**
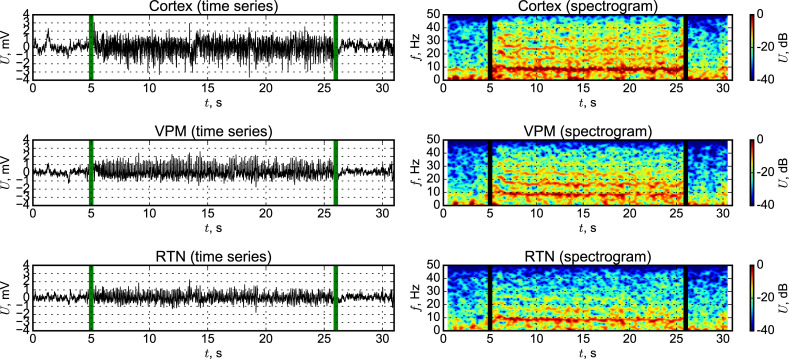
Time series and spectrograms of LFPs from WAG/Rij rats. Three channels are considered: somatosensory cortex, layer 4, ventral posteromedial thalamic nucleus (VPM) and reticular thalamic nucleus (RTN).

**Fig 8:**
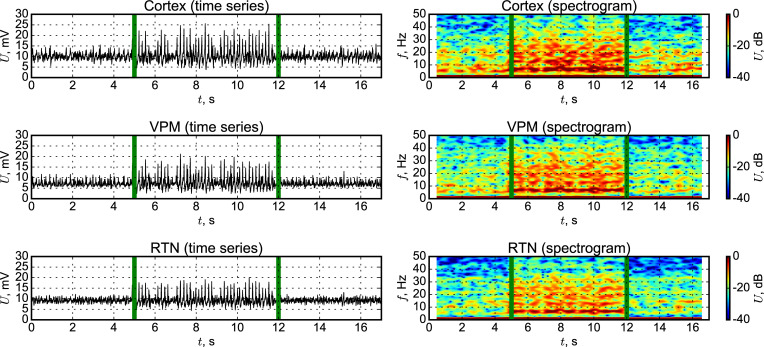
Time series and spectrograms of LFPs from DMM. Somatosensory cortex signals represented by a summary signal from “PY” and “IN” nodes, VPM signal is represented by sum of TC node activities and RTN signal is represented by sum of RE node activities.

Time series and spectrograms of DMM are plotted in Fig 7 The current model reproduces all mentioned properties of LFP signals and their spectrograms associated with transition to SWD, in particular the increase of amplitude and presence of frequency characteristics typical for SWDs with its higher harmonics. Absolute values of LFP signals and zero mean in Fig 8 are the result of amplification and shift by measuring device, so they cannot be considered as reference value for our model. When the spikes during the SWD are compared, one can see that both in our model and in experimental signals the amplitude of cortical ECoG activity, representing extracellular fields, is higher than in the signals from the thalamus. Also, both model and experimental discharges do not look strictly periodic, and demonstrate losses of spikes in some cases and a rather large modulation of the amplitude of the spikes across time.

#### Comparison of model LFPs and single units to those of GAERS rats

Most of the researchers involved in absence epilepsy do not consider that the models (GAERS and WAG/Rij rats) to be very different from each other, both are derived from Wistar rats, although there are differences between the two strains regarding the epileptic genes. In GAERS, a mutated gene has been discovered, which is not mutated in WAG/Rij rats. The fact that different genetic causes might underlie the same epileptic phenotype, is not uncommon. The cells in cortical layer V in the facial area of the somatosensory cortex were found to be excitable, and these cells are thought to be the cause of the SWDs in GAERS [37]. Although the authors of [61] emphasize that there are differences in WAG/Rij and GAERS rats, the main message is that the SWDs are rather similar. Moreover, the site of the focus is the same (facial area of the somatosensory cortex), and the same drugs increase or decrease SWDs in GAERS and in WAG/Rij rats [63]. We do not have intracellular recordings from GAERS in our labs, instead we used published data [64, 65].

**Fig 9:**
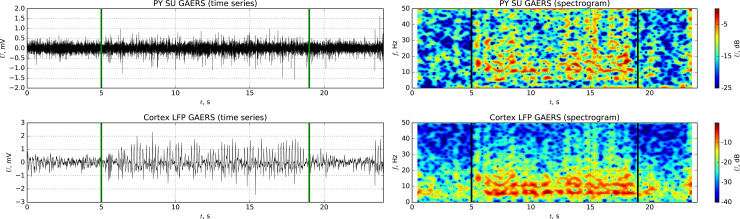
Time series and spectrograms of GAERS rats from the ECoG activity recorded from the somatosensory cortex. Activity of a single unit (pyramidal cell) is plotted at the subplot a, and LFPs are plotted at the subplot b.

Time series and spectrograms of activity of a single node (a) and local field potentials from the surrounding area (b) experimentally measured for GAERS rats are shown in Fig 9. One can see synchronized rhythmic activity during SWD with a main frequency of about 6 Hz (slightly lower due to the neurolept anesthesia) and the presence of its higher harmonics. The firing of cells preictally is irregular. These data support the experimental outcomes of [51], where it was shown that the same cell can demonstrate regular bursting during SWD and irregular activity at other time intervals. The cell activity is noisy due to activities of neighbor cells and currents in the intercellular medium.

**Fig 10:**
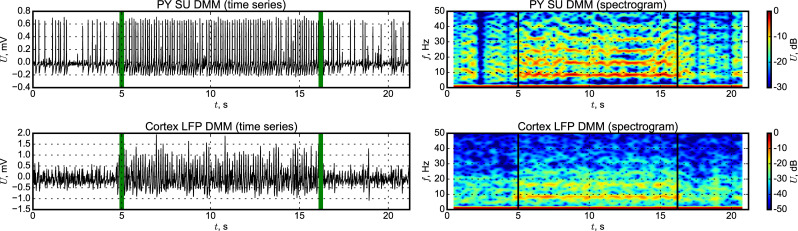
Time series and spectrograms of DMM from somatosensory cortex signal. LFPs (upper plots) are constructed as a sum of signals from all “PY” and “IN” nodes, and single unit signal was taken from a “PY” node.

The model represents a similar pattern (see Fig 10): irregular spikes interictally and regular firing ictally (some spikes are “lost” and that can be seen also in the experimental cell). We have to notice that there is small “bridge”—a number of connections from the “epileptic” (focus) submatrix to the “normal” (surrounding) one, see the rectangular (PYf–column, PYs–row) in Fig 2. These connections almost do not affect the activity of the normal matrix in the background, since first, they are relatively few in comparing with the number of interconnections inside the focal submatrix, and second, the epileptic part is also smaller than the “normal” one. But when SWDs are initiated in the focus, this small “bridge” is enough to include part of nodes from surrounding areas into oscillations. The node, which dynamics is shown in Fig 10, is a part of surrounding matrix but gets the input from another node belonging to the focal submatrix.

### Coupling analysis of model data with Granger causality method

An adapted nonlinear Granger causality method was used here, which was proposed specifically for studying SWDs [66]. The method is based on the construction of empirical predictive models in the time [67], but using non-linear models with specially selected parameters. We evaluated several sets of parameters that met the criteria from [66, 68] in order to find the optimal set of sensitivity / specificity ratio. As a result, the prediction length *τ* = *T*/12 was empirically chosen for both experimental and model data, where *T* is the characteristic oscillation period, a quantity inversely proportional to the main oscillation frequency (i. e., for the absence discharges *T* = 8 Hz). The length of the moving window was chosen *w* = 1 s. The remaining parameters were selected automatically by the BIC criterion [69]: the dimension of the individual model was set to *D*_*s*_ = 4, the polynomial order—*P* = 2, the lag in the model—*l* = *T*/24.

**Fig 11:**
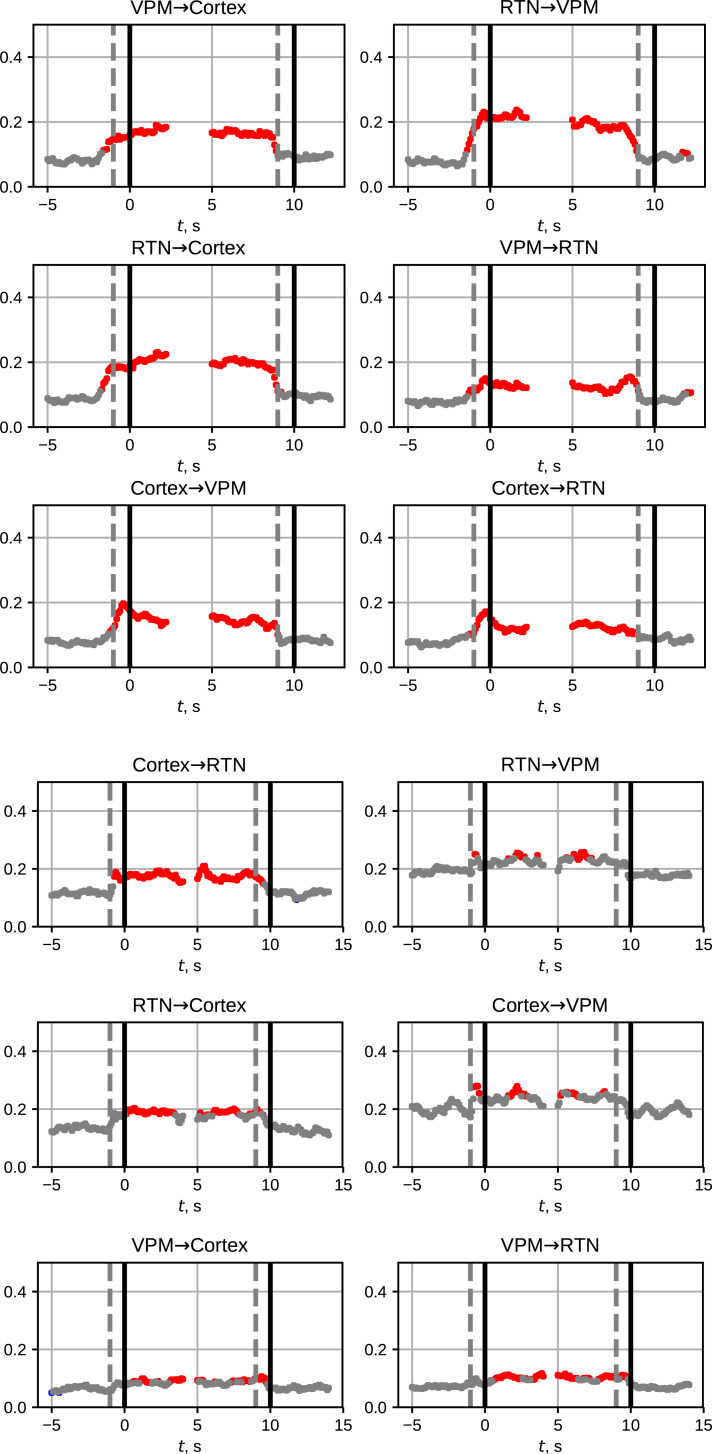
Coupling analysis using the adapted Granger causality method. The subplot a corresponds to LFPs recorded from WAG/Rij rats, and the subplot b corresponds to LFPs generated by DMM. The figure shows the studied intervals before, during and after the seizure (the seizure onset and the seizure termination are marked by black vertical lines). The distance between the black vertical dashed and solid lines indicates the length of the moving window. Gray points show the insignificant average pairwise prediction improvement *PI*_*mean*_, red points correspond to the *PI*_*mean*_ values significantly higher than the baseline level *PI*_*bl*_ at the significance level of 0.05. In the title of each plot it is written the influence from which system to which was tested (y→x).

For each SWD, in the study of Granger causality, intervals, including background (10–3 seconds before the seizure onset) were analyzed, as well as preictal, ictal, and postictal activity. were used. On the plots (see Fig 11) of the prediction improvement versus time *PI*_*mean*_(*t*), the distance between the black vertical dashed and solid lines indicates the moving window length. Gray points show *PI*_*mean*_ values insignificantly different from zero (i. e. from baseline activity), red points correspond to the *PI*_*mean*_ values significantly higher than the baseline level *PI*_*bl*_ (p< 0.05). In the title of each plot the direction of coupling and name of channels between which the coupling was tested were specified using the y→x notation.

If the changes of *PI*_*mean*_(*t*) began within the window (from the dashed to the solid line), then they may originate from the fact that the moving window captures the transition process from the background to the SWD and, accordingly, they are no longer considered as precursor activity.

There is an increase in coupling for both experimental and model data between all structures studied (Cortex, RTN, VPM) during the SWD. However, there are also some differences: in the experimental data one can see a pre-ictal (up to a dashed line) increase in coupling in almost all channel pairs, first of all when RTN increases its coupling strength to the cortex (RTN → Cortex). For the model, a significant increase in coupling begins exactly at the moment of the onset of SWD. Secondly, for the experimental interictally *PI*_*mean*_ are approximately the same for all channel pairs, for the model data, when studying the effects on VPM, *PI*_*mean*_ values interictally are significantly higher than in other channel pairs.

## Discussion

Here a new mathematical model for SWDs at absence epilepsy was provided. In this model we followed the main idea of our previous paper [35] to make a mesoscale model that is able to reproduce most effects (both regarding SWD characteristics and transitional effects from normal activity to SWDs and back) due to coupling organization in the ensemble, not due to individual properties of nodes. The main differences of the current model from the previous one [35] and other models reported in literature are as follows.

The model consists of relatively large number of model nodes (500) in comparison to neuron mass models such as proposed in [7, 70], where only four nodes were considered. This led to the significantly larger model complexity (1000 equations) comparing to [7] where 23 equations were used and [18] with 4 equations. These 500 nodes were structured in comparison to a similar network model for dysrhythmia [33] and organized into five compartments, modeling two thalamic nuclei (VPM and RTN), two cortical node types (pyramids and interneurons), and external input (n. trigeminus) with four of these compartments (excluding that for n. trigeminus) being divided into two parts: focal and surrounding areas; while previously in [23] the model nodes were split only into two (thalamus and cortex) compartments. Inhibitory and excitatory connections were considered following [7], previously this distinction was not made by us. Small time delays in coupling were introduced into the model since axon conductivity takes a reasonable time, so the model has to be considered as a time delayed differential equations model (not ordinary differential equations), having formally an infinite number of degrees of freedom.

The changes in the current model in comparison to the previously known ones, led to the successful modeling of a number of new effects, matching the objectives formulated at the model design. In particular:

1. The model worked as a dynamical system. Adding noise to the model was no longer necessary, and all effects (seizure initiation or termination) induced by changes in the input matrices or due to external stimulation were reproducible. This gave us the possibility to investigate mechanisms of seizure termination by high frequency stimulation, since in the proposed model it is known how SWDs would develop without stimulation. The model correctly simulates SWD termination as a consequence of high frequency 1 s stimulation, in agreement with experimental data [40]. Similar results were already demonstrated using a neural mass model for hippocampal seizures [71], however this could be possible also due to the hippocampal seizures are focal and not initially generalized as SWDs. Therefore, the network was not so necessary as for absence seizures.
2. Three different mechanisms of seizures initiation reported in the literature were reproduced in the model: First, by a short increase of intracortical excitability, second, by an increase of coupling from external input (n. trigeminus), and third, by low frequency (8 Hz) stimulation. All of them led to similar SWDs. All these mechanisms are network mechanisms based on relatively small and short changes in amplitude and by short changes in coupling strength between only a small number of nodes within the focal area. No manual changes of individual nodes parameters or coupling architecture were necessary.
3. Most known models including [7, 18] consider the interictal dynamics and SWDs as two coexisting attractors, with switches between them being possible due to noise. Epilepsy as a multistate phenomenon was considered and discussed based on experimental data simulation in a recent review [70]. In our model SWDs are not considered as an attractor, but as a long transient process (due to system dimension this cannot be proved analytically), and SWD termination is at the same time both deterministic and spontaneous following basic ideas of dynamical chaos. We do not consider this as a model disadvantage, since in dynamical systems of very large dimension like the proposed one, many long living transient phenomena were found, including chimeras [54] and heteroclinic orbits [55, 56] which can work as models for real world phenomena; in particular they were found in networks of neuron models.
4. We were able to compare model time series of both local field potentials and individual nodes to the experimental ones, while in neuron mass models only LFPs could be compared. All main temporal, amplitude and frequency properties of SWDs by themselves and in comparison to interictal dynamics were reproduced. This was proven by comparing model series and spectra to experimental recordings from the most commonly used genetic absence epilepsy models, rats of the WAG/Rij and GAERS lines.
5. An additional process for seizure maintenance was added following [39] to get the mean SWD length close to the length reported in experiments. This approach yielded a probability distribution of SWD lengths very close to known in literature for WAG/Rij rats.
6. The model reproduced most characteristics of coupling dynamics measured from time series [41, 42] for pairs of channels commensurable to the LFP channels provided by the model.

The proposed model was developed for genetic rats and therefore lacks some features of human models. Destexhe’s model of absence seizures [5] generates 3 Hz SWDs, typical for e.g. childhood absence epilepsy. There is also a “rodent” version of this model [72], which generates SWDs of 8 Hz, typically seen in the genetic rat models. These models are biophysically realistic, in the sense that they consider biophysical models of the intrinsic currents and synaptic receptors present in the circuit. Their model presents a number of important features:

1. the seizure occurs when the cortex is made more excitable, with an intact thalamus. This seems relevant to experiments showing that there is indeed an increase of cortical excitability in the genetic rodent models [46] and that an intact thalamus is imperative for the occurrence of SWDs;
2. the model proposes also an explanation why the rats’ SWDs have a frequency of around 8 Hz, while it is typically around 5–6 Hz in cats, and 3 Hz humans.

The mechanism is proposed to be dependent of the relative strength of GABA(A) and GABA(B) conductances in the thalamus. Our model does not differ between GABA(A) and GABA(B) currents (the model of an individual node is not detailed enough for this), therefore our model cannot be used to investigate the contribution of these conductances which could play a role in the difference in frequency between rodents and people. Many different cortical cell layers interact differently during spike and waves as was established in GAERS [73], others showed preictal intracortical processes in WAG/Rij rats [74]. Future models might indeed require different populations of cells in different cortical layers, also since it was found that the superficial layers of the somatosensory cortex are indispensable for the occurrence of SWDs, while the deeper layers communicate directly with the thalamus [75]. Also, it would be interesting to evaluate the predictive validity of future models regarding the effects of locally infused GABA-ergic drugs as well as glutamatergic ones.

In the proposed model all nodes corresponding to different cell types are modeled by the same equations. Many different cortical cell layers interact differently during spike and waves as was established in GAERS [73], others showed preictal intracortical processes in WAG/Rij rats [74]. Future models might indeed require different populations of cells in different cortical layers, also since it was found that the superficial layers of the somatosensory cortex are indispensable for the occurrence of SWDs, while the deeper layers communicate directly with the thalamus [75].

## Supporting information

S1 File (TXT)
